# Comprehensive genomic analysis reveals molecular heterogeneity in pediatric ALK-positive anaplastic large cell lymphoma

**DOI:** 10.21203/rs.3.rs-4145750/v1

**Published:** 2024-03-28

**Authors:** Timothy I. Shaw, Stanley Pounds, Xueyuan Cao, Jing Ma, Gustavo Palacios, John Mason, Sherrie Perkins, Gang Wu, Yiping Fan, Jian Wang, Xin Zhou, Alyssa Obermayer, Marsha C. Kinney, Jacqueline Kraveka, Thomas Gross, John Sandlund, Jinghui Zhang, Charles Mullighan, Megan S. Lim, Vasiliki Leventaki

**Affiliations:** 1 Department of Computational Biology, St. Jude Children’s Research Hospital, Memphis, TN; 2 Department of Biostatistics, St. Jude Children’s Research Hospital, Memphis, TN; 3 Department of Health Promotion and Disease Prevention, University of Tennessee Health Science Center, Memphis, TN.; 4 Department of Pathology, St. Jude Children’s Research Hospital, Memphis, TN; 5 Department of Immunology, St. Jude Children’s Research Hospital, Memphis, TN; 6 Department of Pathology, University of Utah Health Sciences, Salt Lake City, UT; 7 Department of Pathology and Laboratory Medicine, University of Texas Health Science Center, at San Antonio, San Antonio, TX; 8 Division of Pediatric Hematology-Oncology, Medical University of South Carolina, Charleston, SC; 9 Department of Pediatric Hematology-Oncology, Nationwide Children’s Hospital, Columbus, OH; 10 Department of Oncology, St. Jude Children’s Research Hospital, Memphis, TN; 11 Department of Pathology and Laboratory Medicine, Memorial Sloan Kettering Cancer Center, New York, NY; 12 Department of Biostatistics and Bioinformatics, Moffitt Cancer Center, Tampa, FL.; 13 Department of Hematopathology, The University of Texas MD Anderson Cancer Center, Houston, TX

## Abstract

Anaplastic large cell lymphoma (ALCL) is a mature T-cell lymphoma that accounts for for 10–15% of childhood lymphomas. Despite the observation that more than 90% of pediatric cases harbor the anaplastic lymphoma kinase (*ALK)* rearrangement resulting in aberrant ALK kinase expression, there is significant clinical, morphologic, and biological heterogeneity. To gain insights into the genomic aberrations and molecular heterogeneity within ALK-positive ALCL(ALK+ ALCL), we analyzed 46 pediatric ALK+ ALCLs by whole-exome sequencing, RNA-sequencing, and DNA methylation profiling. Whole-exome sequencing found on average 25 SNV/Indel events per sample with recurring genetic events in regulators of DNA damage (*TP53, MDM4*), transcription (*JUNB*), and epigenetic regulators (*TET1, KMT2B, KMT2A, KMT2C, KMT2*E). Gene expression and methylation profiling consistently subclassified ALK+ ALCLs into two groups characterized by diferential ALK expression levels. The ALK-low group showed enrichment of pathways associated with immune response, cytokine signaling, and a hypermethylated predominant pattern compared to the ALK− high group, which had more frequent copy number changes, and was enriched with pathways associated with cell growth, proliferation, metabolic pathways, and. Taken together, these findings suggest that there is molecular heterogeneity within pediatric ALK+ALCL, predicting distinct biological mechanisms that may provide novel insights into disease pathogenesis and represent prognostic markers.

## Introduction

Anaplastic large cell lymphoma (ALCL) is a peripheral T-cell lymphoma that accounts for 10–15% of all childhood lymphomas ^1, 2^. ALCLs exhibit a broad morphologic spectrum characterized by infiltrates of pleomorphic cells and expression of CD30 antigen ^3, 4^. ALCL is divided into ALK-positive (ALK+) and ALK-negative (ALK−) based on the presence of the translocation involving *ALK* ^3*,* 5^. More than 90% of ALCL in children carry a translocation of the *ALK* gene at the 2p23 locus. In ALK+ ALCL, nucleophosmin 1 (*NPM1*) is the major fusion partner, which is described by the translocation t(2;5)(p23;q35)^6^. The translocation results in the expression of a constitutively active oncogenic ALK kinase, which activates multiple downstream signaling pathways, driving malignant transformation and growth of the tumor cells ^7–9^.

ALCL is an aggressive lymphoma, and most pediatric patients with ALCL present with advanced-stage disease ^10^. Even though the standar treatment of newly diagnosed patients with ALK+ALCL is anthracycline-based combination chemotherapy, the most effective and safe treatment for children with advanced ALCL remains to be established^11 12^. While several therapeutic strategies have been explored in clinical trials for pediatric ALCL, they have not led to significant improvement in event-free survival (EFS), with approximately 30% of patients continuing to experience disease progression or recurrence within two years of diagnosis ^10, 13–19^. Notably, clinical, morphologic, and biological factors are associated with prognosis and risk of treatment failure, such as morphological patterns and the assessment of minimal disseminated disease (MDD) at diagnosis ^18, 20–22^.

Integrative strategies using genomic, transcriptomic, and epigenetic profiling offer complementary opportunities to elucidate novel pathogenic insights, functional modules, and deregulated networks in lymphoma pathogenesis. Although the gene expression, genomic and methylation profiles of peripheral T-cell lymphomas ^23, 24^, including ALCL ^25–27^, have been previously characterized, an integrative approach, specifically focused on pediatric ALK+ ALCL has not been performed. Herein, we describe the genomic landscape of pediatric ALK+ ALCL and define the spectrum of somatic mutations, copy number alterations, and gene expression and methylation patterns within ALK+ALCL to identify molecular mechanisms that contribute to its biological heterogeneity.

## Material and Methods

### Subject cohorts and sample details

ALCL cases were collected with informed consent prospectively under a protocol approved by the St. Jude Children’s Research Hospital Institutional Review Board (IRB). The study cohort comprised of 46 fresh frozen tumor samples obtained from the St. Jude tissue resources core facility (N=10), Children’s Cancer and Leukaemia Group (CCLG) (N=18), and Children’s Oncology Group (COG) (N=18). For cases with available material for reviewed, the diagnosis was confirmed by a hematopathologist (V.L.) according to the revised 4^th^ edition of the WHO classification of hematolymphpid tumors ^28^. Information about the % fraction of neoplastic cells in the specimens was available for 45 out of 46 specimens, and estimated to exceed 40% (ranging from 60% to 100%) as determined based on morphologic and/or immunohistochemical studies (applying ALK1 immunostain) for 27 samples with available tissue sections or provided by the tissue biorepository for the COG samples. Matched germline DNA was available for 12 patients. For the St. Jude samples (n=8), germline DNA was obtained from morphologically and immunohistochemically negative bone marrow biopsies obtained at the time of diagnosis; for the CCLG samples (n=4) matched germline DNA was provided by the tissue bank. The clinical and histological characteristics of the pediatric patients with ALCL are summarized in Table 1, and detailed information of the study with available data per patient is given in Supplementary Table S1.

### Whole exome sequencing analysis

Whole exome sequence (WES) analysis was performed as previously described ^29^. Briefly, putative SNVs and indel variants were detected using SNPdetector ^30^. The reference human genome assembly NCBI Build 37 was used to map all samples. Further evaluation of SNVs and indels was performed by manual review of the BAM files using Bambino ^31^. Non-silent coding variations present exclusively in tumors were considered somatic mutations. For variants derived from non-matching germline samples, we applied the unpaired pipeline as has been described previously ^32^. Non-silent mutations were compared to the Exome Variant Server (EVS) (National Heart, Lung, and Blood Institute Exome Sequencing Project; https://evs.gs.washington.edu/EVS/) and to a database of germline variations identified in the Pediatric Cancer Genome Project ^33^. Novel variants that passed this germline filter were annotated with dbSNP v141 and manually reviewed. Known somatic sequence mutations and genes were annotated by the Catalogue of Somatic Mutations in Cancer (COSMIC) database ^34^ and grouped as putative somatic mutations while others were considered variants of unknown origin. The mapping statistics and coverage for each sample are summarized in Supplementary Table S2.

### Validation of Somatic Variants with targeted captured sequencing (TCS)

Mutations were validated using TCS (n =22 samples; AZENTA Life Sciences) designed to cover SNV and indel variants called by WES data (information about the lise of variant evaluated by TCS is included in supplements table S5 and S4). Raw BCL files generated by the sequencer were converted to FASTQ files for each sample using bcf2fastq v2.19. Sequencing adapters and low quality bases in raw reads were trimmed using Trimmomatic 0.39. Cleaned reads were then aligned to the GRCh37 reference genome using Sentieon 202112.01. Each variant was visualized and inspected manually in the IGV viewer^35^.

Among the 46 patients with WES, TCS was performed on 22 patients with available fresh frozen material for analysis (AZENTA Life Sciences, Indianapolis, IN, USA). A TargetGxOne custome gene panel was developed based on the mutated genes detected by WES in the 22 cases submitted for TCS (Supplemental Table S13). Illumins MiSeq, 2×150bp configuration was used for the sequencing (AZENTA Life Siences). 196 somatic SNV/Indel calls by the WES variant-calling pipeline from 46 patients were covered by the custom capture panel, and 180 out of 196 variants (90.9%) were validated (Supplementary Table S5, S6). For the 36 cases (32 diagnostic and 4 relapsed samples) with RNAseq data, 126 of 132 (95.4%) somatic SNV calls from WES data were validated by RNAseq (Supplementary Table S5). In three cases with the recurrent *JUNB* A282V mutation was also validate by Sanger sequencing (Suppl. Fig3.B).

### Copy number variant analysis using WES data

Copy number analysis was performed using CNVkit version 0.9.1 on the exome sequencing data ^36^. Target and anti-target regions were defined based on Nextera rapid capture region and “access-5k-mappable.hg19.bed”. The copy number reference control was pooled from germline exome sequencing. Due to the inherent noise of the exome-based copy number analysis, for each copy number altered region, we manually visualized the B-allele frequency shift to validate the copy-number alterations (Supplementary Figure S4, B-C).

### RNA sequencing, mapping, fusion detection, and relapse signature analysis

Total RNA was prepared from cryopreserved tumor samples by a single-step RNA isolation method (TRIzol Reagent, Life Technologies), mechanically homogenized with the TissueRuptor (Qiagen), and quantified using the Agilent 2100 Bioanalyzer (Agilent Technologies). Indexed RNA sequencing (RNA-seq) libraries were constructed from 250 ng of total RNA by using the TruSeqTotal Stranded RNA Library Prep Kit (Illumina). Each library was sequenced in a paired-end mode, using 1 lane of the HiSeq 2000 (Illumina) flow cell, which generated 2–3 × 100 bp reads. RNA-seq reads were aligned by using the BWA^37^ (0.5.10) aligner to the following database files: (i) the humanGRCh37-lite reference sequence; (ii) RefSeq; (iii) a sequence file representing all possible combinations of non-sequential pairs in RefSeq exons; and (iv) the AceView database flat file downloaded from the UCSC Genome Browser, which represents transcripts constructed from human ESTs. The mapping results from databases (ii)–(iv) were aligned to human reference genome coordinates, and a BAM file was constructed by selecting the best alignment from the 4 databases. The mapping statistics and coverage for each sample are summarized in Supplementary Table S3. Structural variations were detected by using CICERO, a novel algorithm that uses *de novo* assembly to identify structural variation in RNA-seq data ^38^. The *ALK* fusion transcript was manually reviewed by using the Bambino viewer ^31^ and the Integrated Genomics Viewer ^39^. Mapped reads were counted with HTseq ^40^ and normalized to FPKM (fragments per kilobase of transcript per million mapped reads) (Supplementary Table S4).

To generate the gene expression signature of relapsed ALCL, differential gene expression analysis was performed comparing relapse (n = 4) vs. diagnosis (n = 32) samples. Genes significantly up-regulated in relapsed samples (P < 0.01, Log2FC > 1, and adjusted p-value < 0.25) were used as the gene set for single-sample gene set enrichment analysis (ssGSEA). Gene expression pathway analysis was performed using GSEA ^41^. The Hallmark, KEGG and Oncogene databases were used for GSEA. Genes were ranked using signal2noise with 1000 permutations performed on the gene set. xCell was applied to define the immune infiltration ^42^.

### DNA methylation analysis

Genomic DNA (1 μg) from 37 ALCL was bisulfite converted by using the EZ DNA Methylation kit (Zymo Research Corp, Irvine, CA, USA). Converted samples were processed and hybridized to the Infinium MethylationEPIC BeadChip (850K) system (Illumina, San Diego, CA, USA). The methylation score of each CpG site in the array is represented as a beta (*β*) value and was computed using the methylation module of the GenomeStudio software (version 1.9.0; Illumina). Beta-values were converted to M-values ^43^ prior to differential methylation analysis and clustering. Each probe was correlated to the ALK gene expression by the Spearman rank correlation. An absolute rho value of 0.75 was used as the cutoff to identify methylated genes for downstream pathway analysis by ENRICHR. The differentially methylated regions were characterized by the Wilcoxon Test with Benjamini Hochberg (BH) Adjusted Pval < 0.05. Probes residing in chromosome X and Y and with SNP VAF > 0.01 were removed from the analysis.

### Cluster analysis of RNAseq and DNA methylation data

Clustering for both RNAseq and methylation array data was performed by calculating the top variable genes/probes using MAD, Variance, and DIP. Bootstrap hierarchical clustering was performed using a combination of “complete” and “average” hierarchical clustering algorithms. To determine the number of features that produce a stable hierarchical clustering assignment, we calculate the bootstrap probability while steadily increasing the optimal number of features. The cluster assignment was then compared to the top 1000 variable gene/probe’s cluster assignment. Finally, we took the consensus across the different methods for the final group assignment. Differential gene expression analysis was performed by LIMMA ^44^ using log2 fold chang (log2FC) > 1.0 and FDR adjusted p-value < 0.05 as cutoffs.

### Statistical methods

The student’s t-test, two-tailed, assuming equal variances, and Wilcox signed-rank test were used to compare two experimental groups. The Fisher’s exact test was used to test for the consistency of the subgroup assignment by RNAseq and methylation. Overall survival and progression-free survival were defined as the time difference between the date of diagnosis and the date of death or relapse. Patients who were alive at the time of the last follow up were considered censored. Survival curves were compared via log-rank tests.

### Data availability and accession codes

Genomic data have been deposited in the European Genome-phenome Archive (EGA), which is hosted by the European Bioinformatics Institute (EBI), under accession EGAS00001004189. The accession number for the Illumina methylation reported is GEO: GSE186487. All other remaining data are available within the Article and Supplementary Files.

## Results

### Sequencing of pediatric ALK+ ALCL

**Figure 1.**
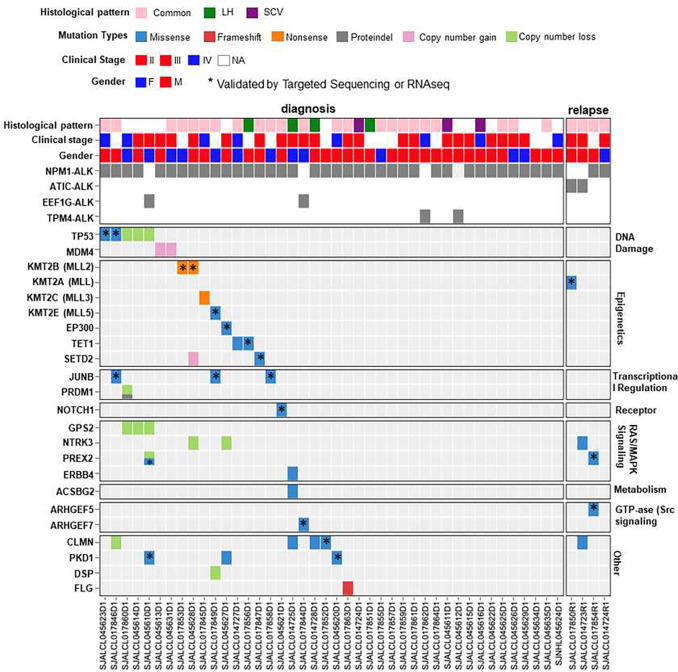
Genomic alterations in pediatric ALCL. Heatmap demonstrates the somatic mutation profile and copy number (CN) alterations identified in the cohort of pediatric ALK+ALCL samples by WES, separated by gene functional groups. Only genetic alterations with presumed functional consequences are shown. Split cell for the *PRDM1* gene indicates more than one mutation. (*) indicates mutations validated by targeted sequencing or RNAseq.

We performed next-generation sequencing on a cohort of 46 pediatric ALK+ ALCLs, including 42 tumor samples obtained at diagnosis and four relapsed samples (1 patient with paired diagnostic-relapsed samples) (Supplementary Figure S1.). The histological pattern, defined as common, lymphohistiocytic (LH) or small cell variant (SCV), was available for a subset of patients (34 out of 46) in our cohort. For the remaining cases the subtype was not included in the pathology report or a tissue section available for morphologic assessment was not sufficient to define the histological pattern with confidence. RNA sequencing was performed on 44 samples with available material; however, 10 samples failed quality control and were excluded from the analysis. For all the cases, the presence of the *ALK* fusion was confirmed by the RNA sequencing data, and for cases with available tissue material, the expression was also verified by ALK1 immunohistochemistry. RNASeq analysis confirmed that the *NPM1-ALK* was the most common fusion identified in 40 out of 46 patient samples (Figure 1; Supplementary Table S8). Remaining ALK+ patients were characterized by *ATIC::ALK* (n = 2), *TPM4::ALK* (n = 2), and *EEF1G::ALK* (n = 2). The two patients with a novel *EEF1G::ALK* fusion were previously published by our group ^45^.

Whole exome sequencing (WES) was performed on all cases with a median coverage of 92X in the tumor (Supplementary Table S4). Matched germline DNA was available for 12 cases. Sample read coverage varied from 36 to 198 million reads with an average of approximately 120 million reads per sample, and ≥96% reads were mapped to the reference human genome (hg19). For the 46 cases, the WES for the 12 samples with paired germline DNA identified a total of 238 SNVs and 18 indels*.* The somatic mutation rate was estimated to be 25.6 events per sample in paired germline specimens. For the 34 samples with tumor-only material, mutations were filtered to exclude silent and UTR mutations, resulting in a total of 300 amino acid variants (192 SNVs and 108 indels; Supplementary Table S5, S6, S7). Next, we compared the mutation burden of the ALCL samples to other pediatric malignancies analyzing WES data from the St Jude Pediatric Genome Project ^46, 47^. The mutational burden in pediatric ALCL samples was higher than the mutational burden in pediatric acute leukemia but lower compared to most common pediatric solid tumors, such as adrenocortical carcinoma, osteosarcoma, rhabdomyosarcoma, and high-grade glioma (Supplementary Figure 2B).

In Figure 1, we highlighted genes with genomic alterations that (i) were identified in more than 2 cases, (ii) validated by RNA seq, (iii) are known to have biological significance in cancer, and (iv) have been previously shown to be altered in lymphoma ^48, 49^. 25 out of 46 patient samples observed somatic events (SNV, CNV) in genes associated with DNA damage, epigenetic and transcriptional regulators, metabolism, Rho-family GTPases, and RAS-MAPK signaling pathways. Mutations in epigenetic regulators (*EP300, KMT2A, KMT2D, KMT2C, KMT2E, TET1, SETD2*) were observed in 9 out of 46 cases. Genomic alterations of components of the JAK/STAT pathway, which frequently occurs in ALK− ALCL ^25^ were absent in our cohort. Two cases contained single nucleotide mutation in *TP53*, affecting the DNA binding (R280K) and tetramerization domains (Q331H). Three out of 46 cases demonstrated a recurrent *JUNB* mutation (A282V) (Supplementary Figure 3A). This mutation affects the conserved DNA binding domain of JUNB and has been recently described in three cases in a cohort of ALK− ALCL^50^. JUNB is a member of the AP-1 transcription factors, and has been shown in previous studies to regulate tumor growth and proliferation in ALK+ALCL ^50–53^. ^50^. The mutation was validated by RNAseq in all three cases but with a higher VAF possibly indicating that there is a preference for the expression of the mutated allele (Supplementary Figure 3B). Of note, all the cases with the *JUNB* mutation showed similarly high tumor content.

Copy number analysis detected 45 genetic alterations in 13 out of 46 ALCL tumors (Supplementary Table S9; Supplementary Figure S4A) and validated by B-allele frequency (Supplementary Figure S4B, S4C). CN alterations included losses of 17p13.1 (3/46 samples), which encompasses *TP53* and *GPS2*, 6q21/*PRDM1* (one sample with both indel and copy number loss), and 10p4/*GATA3* (2 samples), and gains of different regions of chromosome 1q, including the region of 1q32.1/*MDM4* (2 samples) and 1q44/*AKT3* (2 samples). In agreement with previous studies ^54, 55^, *TP53* alterations were infrequent in our pediatric cohort. Collectively, there were five cases (5/46; 11%) with genomic alterations affecting *TP53* and these did not overlap with the two cases that showed gain of 1q23.1/*MDM4*.

### Gene expression signatures and methylation profiling identify two subgroups of pediatric ALCL

**Figure 2.**
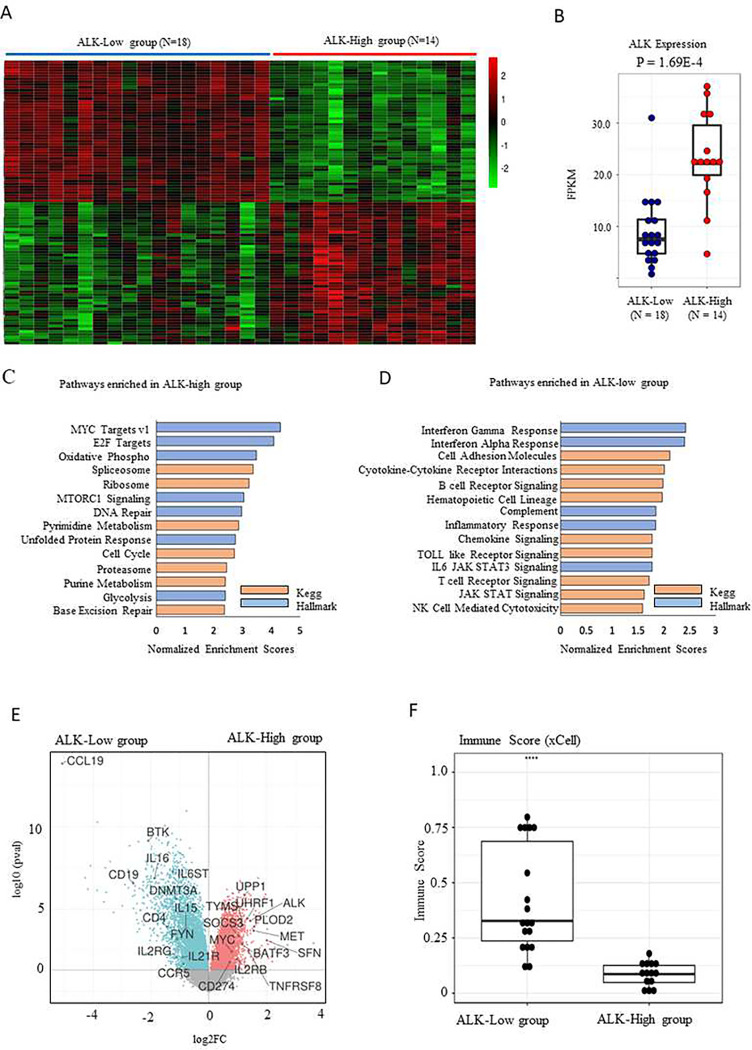
Gene expression profile classifies pediatric ALCL into two groups. **(A)** Unsupervised hierarchical clustering reveals two distinct groups. The top 100 differentially expressed genes are shown as a heatmap. **(B)** Boxplot of differential ALK expression levels in ALCL samples detected by RNA seq analysis (*P*=0.026). FPKM, fragments per kilobase of exon per million mapped fragments. **(C and D)** Pathway analysis in ALK-low and ALK-high groups (KEGG and MSigDB), p-value <0.05 and FDR < 0.05. **(E)** Differential expression represented as a volcano plot. ALK-low group shows up-regulation of cytokine and immune related markers. ALK-high group shows up-regulation of ALK, MYC, and other proliferative markers. **(F)** xCell analysis shows differential Immune Scores in ALCL samples.

To identify distinct molecular signatures and to assess potential molecular heterogeneity within ALK+ALCL, we performed an unsupervised clustering analysis of RNAseq data from 32 diagnostic samples, excluding the four relapsed samples (Supplementary Table S1 and Figure S1). Two distinct subgroups of patients were identified with greater than 70% bootstrapping support across clustering methods (Figure 2A; Supplementary Table S4). The two groups could be defined based on differential ALK expression levels into ALK-high (n=14) and ALK-low group (n=18) (Figure 2B) ^56, 57^.

Among the differentially expressed genes, 2878 were up-regulated, and 3008 were downregulated in the ALK-high group when compared with the ALK-low group. Gene set enrichment analysis using the MSigDB, and KEGG databases found enriched pathways of cell proliferation, growth (MYC, E2F, and MTORC1), pyrimidine/purine metabolism, and glycolysis in the ALK-high group (Figure 2C). Upregulated genes in the ALK high group included *UHRF1*, a critical regulator for maintaining DNA methylation ^58, 59^, and the immunoinhibitory molecule, CD274 (*PD-L1)* ^60^(Figure 2E). Genes up-regulated in the ALK-low group included the epigenetic regulator, *DNMT3A,* and genes involved in cytokine-cytokine interaction, including *CCL19*, ligand of CCR7 ^61^, and interleukin signaling, such as *IL16, IL21R*, and *IL2RG* ^62^ (Figure 2E). Overexpression of the components of the IL2R has been previously shown in ALK+ALCL cell lines and patient samples as we and others observed in the ALK-low group^56, 63^. Consistently, pathways enriched among ALK-low samples related to immunologic functions, including interferon response, B-cell receptor signaling, cytokine-cytokine receptor interactions, and chemokine signaling pathways (Figure 2D). Using the xCell algorithm, the ALK-low patient samples were characterized by an overall higher immune score compared to the ALK-high group (Figure 2F; Supplementary Figure S5A; T-Test p-value < 0.001; Supplementary Table S11), and higher numbers of immune cells such as B-cells, activated dendritic cells, and T-cells (Supplementary Figure S5B-D).

**Figure 3.**
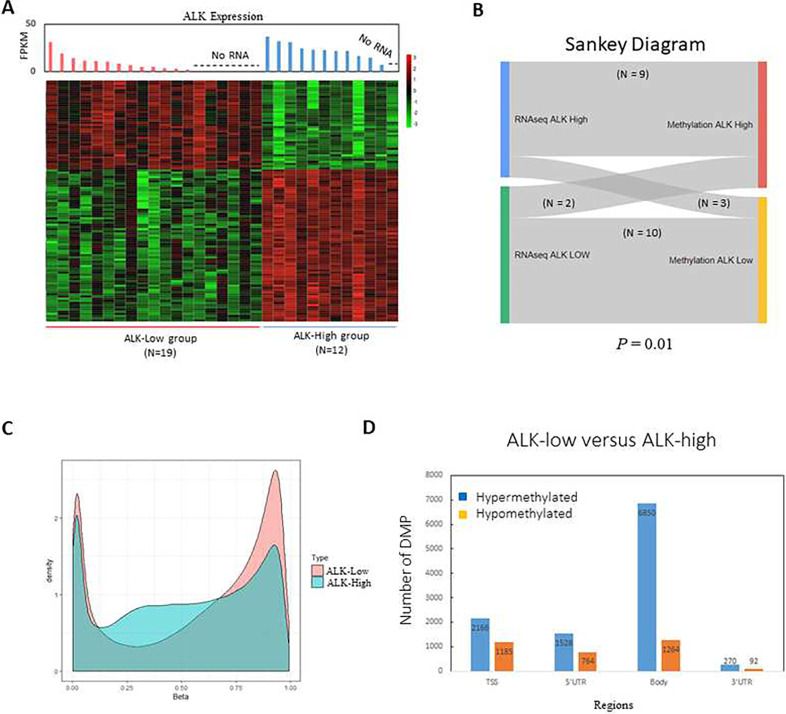
Genome-wide DNA methylation classifies pediatric ALCL in two groups. **(A)** Unsupervised clustering with bootstrapping identified two separate clusters. Each probe was then summarized to a single value for each associated genomic region. Each methylation value was annotated as Hyper-methylation or Hypo-methylation. Differential methylation was performed using Wilcoxon rank-sum test. FPKM, fragments per kilobase of exon per million mapped fragments. **(B)** Sankey diagram representation of the unsupervised hierarchical clustering result. The diagram depicts the group clustering based on RNA expression and methylation profiling. **(C)** Histogram of the methylation probe intensity (Beta value) in the ALK-high and ALK-low group **(D)** Bar plot representation of the differentially methylated gene regions. Hyper-methylated regions were more frequently differentially methylated than hypo-methylated regions. DMP, differentially methylated positions; TSS, transcription start site.

In addition to gene expression analysis, we identified distinct groups of pediatric ALCL using DNA methylation array profiling, an approach that has been extensively used to define molecular subgroups in tumors of the central nervous system ^64–66^. After excluding cases with <40% tumor content, DNA methylation analysis was performed on 31 diagnostic samples (Supplemental Figure S1). Unsupervised clustering revealed two distinct clusters with greater than 70% bootstrapping support (Figure 3A). For the twenty-four diagnostic samples analyzed by both DNA methylation and RNAseq, 19/24 samples (79%) had a consistent ALK-high or ALK-low group assignment (p-value < 1.96E-3; Figure 3B). For seven samples analyzed by DNA methylation only, six samples were grouped with the ALK-low group, and one sample was grouped with the ALK-high group. The DNA methylation pattern in ALK-low samples resembled a bimodal distribution. ALK-high samples contained moderate levels of methylated probes with a beta value ranging from 0.33 to 0.66 (Figure 3C). More hypermethylated regions were found in the ALK-low samples, particularly in the gene body (6850 hypermethylated, 1264 hypomethylated) and transcription start site (TSS) region (2166 hypermethylated, 1385 hypomethylated) (Figure 3D). This finding is in line with the observation of DNTM3a overexpression in the ALK-low group that may contribute to its hypermethylation profile. The association between DNTM3a and highly methylated genome affecting mostly CpG islands has been described in T-lymphoblastic leukemia^67^. Interstingly, Hassler et al described a differential methylation pattern between ALK− and ALK+ ALCL tumors mainly based on hypomethylating MVPs in the ALK+ tumors^27^ that supports our observation of a more hypomethylated profile in the ALK-high group compared to the ALK-low group.

**Figure 4.**
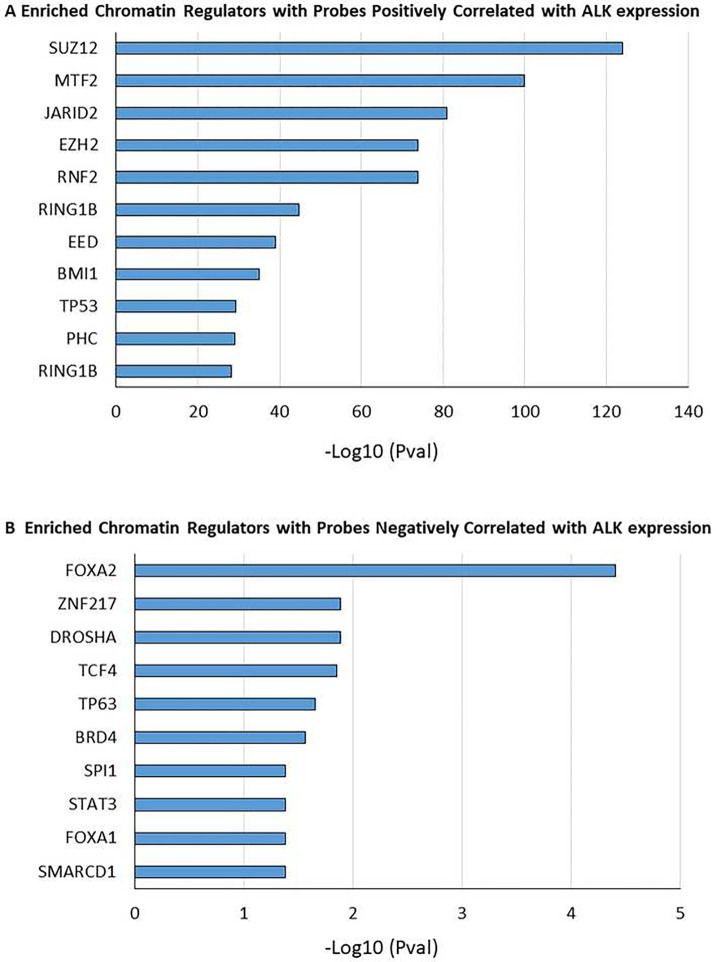
Genes that show correlation between probe methylation status and ALK expression Correlation analysis is based on Spearman Rank with positive correlation shown in **(A)** and negative correlation shown in **(B).** Both are filtered based on an absolute rho value cutoff of 0.75. Bar plots show enriched pathways from EnrichR’s ChEA_2016 sorted by their log transformed p-value score.

To identify biologically relevant functions and pathways affected by DNA methylation that could be associated with ALK expression, each probe methylation was correlated with the ALK expression levels in the 24 samples with available RNA seq and DNA methylation array data, excluding relapsed samples and samples with tumor content <40%. Methylated regions correlated (positive and negative)with ALK expression were selected for pathway analysis using the ChEA transcription factor database ^68^. Hypermethylated genes that track with high ALK levels (positively correlated), were enriched for the Polycomb-repressor complex 2 (*SUZ12, EZH2*, and *JARID2* complex; Figure 4A). Hypermethylted genes that track with low ALK expression level (negatively correlated) were enriched for the BRD4 and TCF4 chromatin regulators (Figure 4B), which have been shown to induce *MYC* expression ^69, 70^. This finding is consistent with the expression profile of the ALK-high group characterized by increased MYC expression. Prior studies have also described *MYC* amplification as a mechanism of its overexpression that we did not observe in our cohort based on the analysis of WES data for CNA^71, 72^. Altogether, the findings suggest that ALK may contribute to differential DNA methylation patterns between the two groups that may subsequently contribute to reprogramming of transcription and distinct expression profiles.

### Prognostic significance of genetic alterations and group assignment in pediatric ALCL

**Figure 5.**
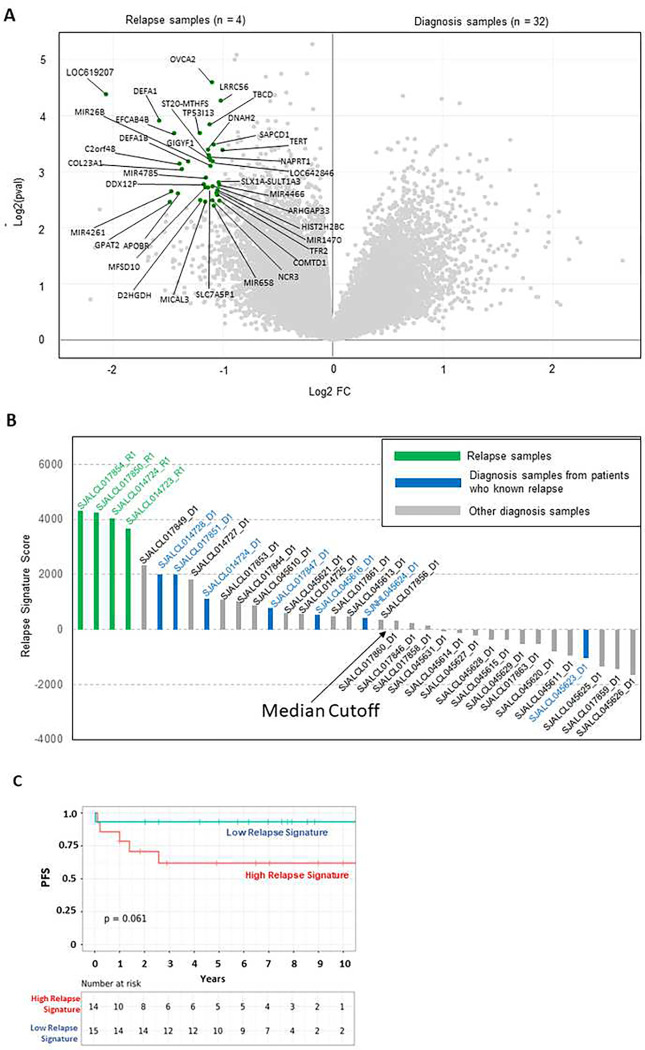
Relapsed signature score in pediatric ALK+ALCL patients. **(A)** Differential gene expression analysis between relapse and diagnosis samples from ALCL patients. Genes differentially up-regulated in relapsed samples are highlighted in green. **(B)** The relapse signature score is calculated based on single-sample GSEA. Samples were ordered based on the calculated relapse signature score. Relapse samples are colored in red. Diagnosis samples of patients who eventually relapse are colored in blue. The remaining diagnosis samples are colored in gray. The median cutoff for the diagnosis samples is indicated by the arrow. **(C)** Kaplan-Meier graph showing progression free survival (PFS) according to the relapse signature score. The relapse signature score was categorized into HIGH and LOW based on the median level. A statistically significant difference of the Kaplan–Meier survival curves between the high and low malignancy-risk groups was determined by the two-sided log-rank test. The number of patients at risk is listed below the survival curves.

To identify molecular events associated with ALCL relapse, differential gene expression analysis was performed comparing four relapsed samples to 32 diagnostic samples. Differential expression analysis revealed 36 genes up-regulated in relapse compared to diagnostic samples (Figure 5A). Gene set enrichment analysis (GSEA) demonstrated that MYC targets and negative regulation of genes involved in the inflammatory response pathways are enriched in the relapsed samples (Supplementary Figure 6A-B). While the relapse-associated genes from Camille et al. ^73^ were not consistently upregulated in our cohort of relapsed RNAseq samples (Supplementary Figure 6C-D), diagnosis-associated genes were consistently up-regulated in our cohort of diagnosis ALK+ ALCL, which were enriched for genes involved in the extracellular matrix region (Supplementary Figure 6E-F). To evaluate the relationship between relapse gene signature and clinical outcome, we developed a relapse signature score by single-sample GSEA and analyzed the RNA sequencing data from our pediatric ALCL cohort (Supplementary Table S12). Interestingly, we found an elevated relapse signature present in the diagnosis sample of patients who eventually relapsed (Figure 5B). Of note, only for one patient (SJALCL014724) in our cohort there were available both a diagnostic and a relapse specimen, showing the relapsed signature. Notably, the diagnostic sample for this patient showed the small cell variant that has been consistently associated with worse prognosis in the pediatric ALK+ALCL. Of clinical relevance, the diagnosis samples with high relapse score (above median) trended toward a worse prognosis (OS, p = 0.056; PFS, p = 0.061; Figure 5C). Overall, these results suggest that the gene expression signature at diagnosis could be predictive of relapse associated with an inferior prognosis in a subset of pediatric ALCL patients.

**Figure 6.**
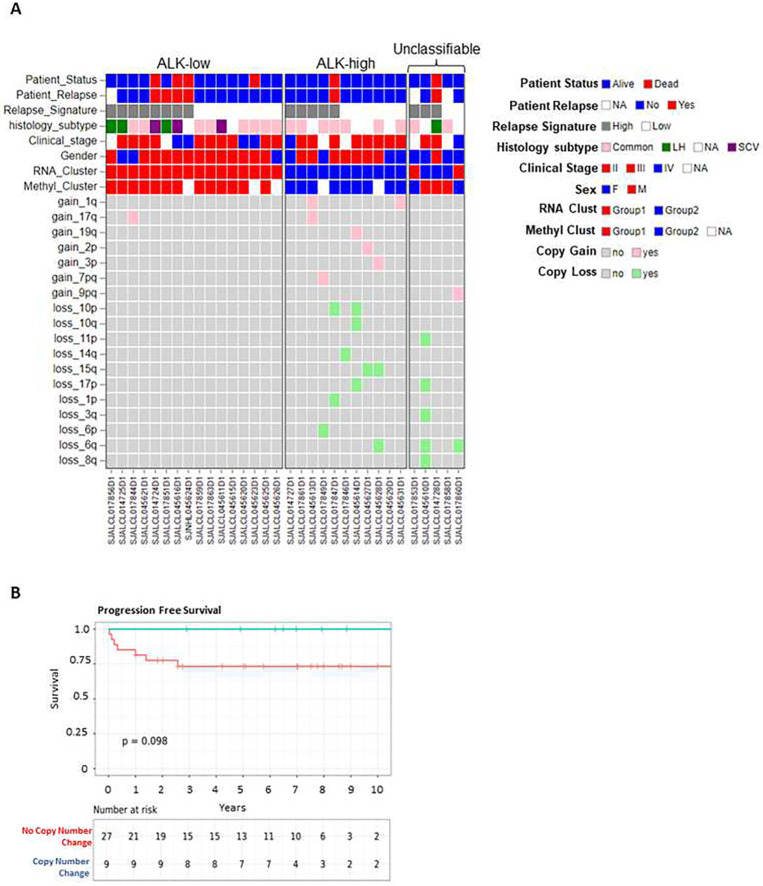
Overview of clinical findings and copy number changes associated with the two ALCL groups and impact on outcome. **(A)** Heatmap with the distribution of clinical findings and copy number alterations in the diagnostic ALCL samples; cases are assigned to ALK-low and ALK-high group based on the expression and methylation profile. Five cases were classified differently based on RNA expression and DNA methylation analysis (designated as unclassifiable) **(B)** Kaplan-Meier curve showing progression free survival (PFS) in ALCL according to the presence of copy number changes.

Next, to investigate the potential prognostic role of group assignment and genetic alterations in our cohort, we grouped the diagnostic only ALCL cases (N=32) based on their expression and methylation classification into ALK-low and ALK-high group. Cases with disagreement in group assignment between expression and methylation profile were designated as unclassifiable. (Figure 6A). CN alterations were enriched in the ALK-high group (72.7%; 8/11) compared to the ALK-low group (6.25%;1/16) (p-value < 0.0198; Figure 6A). Six out of seven cases with the non-common histology (3 cases of LH and 3 cases of SCV) were assigned to the ALK-low group by both expression and methylation analysis. Of clinical relevance, the group of patients without CN alterations trended toward an inferior progression-free survival (*P*=0.098, Figure 6B), which was previously observed in a cohort of systemic ALK+ALCL not strictly pediatric ^26^. However, group assignment in ALK-low or ALK-high based on differential expression and methylation profile did not show an association with clinical outcome (Supplemental Figure 7A-B). Also, due to the sample size, we could not investigate in detail whether specific genomic lesions could stratify patients further regarding prognosis and clinical outcome.

## Discussion

Previous studies on ALCL have focused on identifying genetic alterations in ALK negative ALCL (ALK-ALCL) and gene expression signatures that differ between ALK+ and ALK− ALCL ^57^, supporting their distinction into separate entities. In this study, we sought to define molecular heterogeneity in pediatric ALK+ ALCL (diagnostic and relapsed samples) using an integrated analysis including WES, RNA-sequencing, and DNA methylation array. Unsupervised cluster analysis of the expression data (RNAseq) identified two distinct subgroups of pediatric ALK+ ALCL defined by defferential ALK expression levels, the ALK-high and ALK-low groups, consistent with previous array expression data ^56, 57^. Through our integrative approach, we identified distinct molecular features associated with each of the two subtypes. The ALK-high group was enriched with somatic mutations and copy number changes, including genes associated with chromatin modification, cell cycle, and receptor tyrosine kinase signaling. The ALK-low group was significantly enriched in genes associated with cytokines and immune-related gene-sets. Differentially expression of methylation regulators was also notable between the two groups especially in association with differences in methylation patterns (discussed below), possibly related to DNMT3a overexpression in the ALK-low group, and UFHR1, a regulator of DNMT1, in the ALK-high group. A pattern of hypermethylation especially in CpG islands has been described in a T-ALL that was also correlated with DNMT3B ^67^. Overall, our findings support a molecular heterogeneity within pediatric ALK+ ALCL, while it is uniformly characterized by the transforming activity of the aberrant ALK expression. We found mutations affecting epigenetic regulators that have been described previously in different subtypes of mature T-cell lymphomas ^74, 75^. The functional role of genetic alterations of epigenetic regulators in ALK+ ALCLs is unknown and worth future investigation.

Mutations affecting the JAK/STAT3 pathway, observed in ALK− ALCL cases ^25^, were absent in our cohort of pediatric ALK+ ALCL. This corroborates the existing literature that supports the role of *ALK* fusion as a strong driver oncogene in ALK+ ALCLs. In our cohort of pediatric ALK+ALCL, one case showed *PRDM1* alterations affecting both alleles (indel and CN loss) with concurrent loss of *TP53* loci (17p). Previous studies have shown that loss of *TP53* and *PRDM1*, more frequently observed in ALK− ALCL than in ALK+ALCL cell lines, play a role in the pathogenesis and are associated with a less favorable outcome ^26^. The CN alterations identified in our cohort using the WES data included gains of 1q, and losses of 6q and 17p. These findings are similar to the CNA reported by Boi et al using SNP array analysis in a cohort of ALCL (ALK+ and ALK−) patients^26^, while differing from the genomic imbalances described in earlier studies that used GCH arrays that included gains of 2p, 7p, 17p and 17q, and losses of 4q, 11q and 13q^76^. The different findings between older studies focused on copy number alterations could be most likely related to applying different technical approaches. For our copy number analysis approach using the WES data the main limitation was the lack of paired tumor-germ line DNA for a substantial number of the cases.

Arm level aneuploidy appears to be highly prevalent in ALK-high group, likely resembling aneuploidy and chromosome instability ^77^ but largely absent in the ALK-low group. The ALK-high group revealed up-regulated pathways involved in cell cycle and cancer proliferation, which recapitulates an accelerated tumorigenesis observed in a chromosome instability induced aneuploidy lymphoma model ^78^. Previous studies have also reported similar observations in adult solid tumors, associating somatic copy number change with immune evasion and reduced immunotherapy response ^79^. Our data suggest that this observation is not unique to solid tumors and may be relevant for lymphoma pathogenesis.

We further demonstrated that the molecular heterogeneity within the pediatric ALK+ALCL is supported by differential methylation patterns between the two groups identified by the expression cluster analaysis. ALK expression was associated with methylation of regions/probes harboring repressive histone marks as indicated by the enrichment of chromatin regulators of the PRC2 complex (EZH2, SUZ12, and JARID2), which has been described as “epigenetic switching” in cancer ^80^. Negative correlation between ALK expression and methylation was observed for the chromatin regulators, BRD4 and TCF4, suggesting a role in the regulation of MYC expression that was found to be upregulated in the ALK-high group. These findings provide a rationale for the evaluation of novel targeted therapies such as MYC/bromodomain inhibitors ^81^ and PRC2 inhibitors in ALK+ALCL ^82^. Additionally, this observation suggests that ALK-expression levels may be associated with DNA methylation and gene expression levels.

In our series, there was no statistically significant difference in outcome between the ALK-high and ALK-low groups by differential expression and DNA methylation. However, a tendency for worse outcomes was observed in cases without copy number changes that predominantly included ALK-low samples.

A transcriptional relapse signature was derived from four samples in our cohort that predicted relapse in the diagnosis samples. By comparing our expression profile to Camille et al. ^73^, the down-regulated relapse signature was found consistent between the two datasets but inconsistent in the up-regulated relapse signature. The discrepancy might be due to the relatively limited number of evaluated cases in our cohort, the high frequency (54%) of variant histology in the study by Camille et al. versus 15% in this study, and the heterogeneous nature of different therapies. Confirmation of these findings requires validation on an independent cohort of homogeneously treated pediatric ALK+ ALCL patients.

Our comprehensive, integragrative analysis of WES, RNA-seq, and DNA methylation data provide important features and further insights into the molecular heterogeneity in a strictly pediatric cohort of ALK+ALCL. In addition to supporting the previous observations of two distinct groups in ALK+ALCL based on the expression profile and ALK expression level (ALK-low and ALK-high), we have incorporated genomic (copy number alterations) and DNA methylation data to further characterize the molecular heterogeneity in this entity. Our observation of differentially expressed patterns between the two groups highlighting the role of immune response and proliferation markers, along with differences in the overall methylation profile, may help to focus on further investigation of novel molecular mechanisms that contribute to lymphogenesis, progression and as targeted therapies for children with newly-diagnosed and/or relapsed ALK+ALCL.

## Figures and Tables

**Table 1.** Clinical and pathological characteristics of the pediatric ALCL cohort.

Clinical and biological characteristics		*N=46* ^¶^

Gender	Female	12
	Male	34
Median age at diagnosis (yrs)	≤ 8.5	15
	>8.5	31
Stage at diagnosis	I-III	26
	IV	7
	Unavailable	13
Mediastinal involvement	Yes	17
	No	8
	Unavailable	21
Skin involvement	Yes	5
	No	26
	Unavailable	15
Histological patterns	Common	27
	Other*	7
	Unavailable	12

* Other: other than common patterns, including the small cell variant (SCV) and lymphohistiocytic (LH) variant.

¶ One paired sample (diagnostic-relapsed) is included.
